# Type II cGMP-dependent protein kinase inhibits the migration, invasion and proliferation of several types of human cancer cells

**DOI:** 10.3892/mmr.2022.12679

**Published:** 2022-03-14

**Authors:** Min Wu, Yan Wu, Hai Qian, Yan Tao, Ji Pang, Ying Wang, Yongchang Chen

Mol Med Rep 16: 5729–5737, 2017; DOI: 10.3892/mmr.2017.7290

Subsequently to the publication of this paper, an interested reader drew to the authors’ attention that Figs. 3 (showing how PKG II overexpression inhibits the migration of various types of cancer cells) and 6 (showing representative photomicrographs of apoptotic cells under different experimental conditions at ×200 magnification) contained apparently duplicated data panels within the figures. After having examined their original data, the authors have realized that these figures were inadvertently assembled incorrectly; specifically, the data shown in the HepG2-Ad-LacZ+EGF and OS-RC-2-Ad-LacZ+EGF panels in Fig. 3A and the HepG2-Ad-LacZ+cGMP+EGF, OS-RC-2-Ad-PKGII+cGMP+EGF and U251-Ad-PKGII+cGMP+EGF panels in Fig. 6A were selected incorrectly.

The corrected versions of Figs. 3 and 6 are shown on the next two pages. Note that these errors did not significantly affect the results or the conclusions reported in this paper, and all the authors agree to this Corrigendum. The authors are grateful to the Editor of *Molecular Medicine Reports* for allowing them the opportunity to publish this and apologize to the readership for any inconvenience caused.

## Figures and Tables

**Figure 3.**
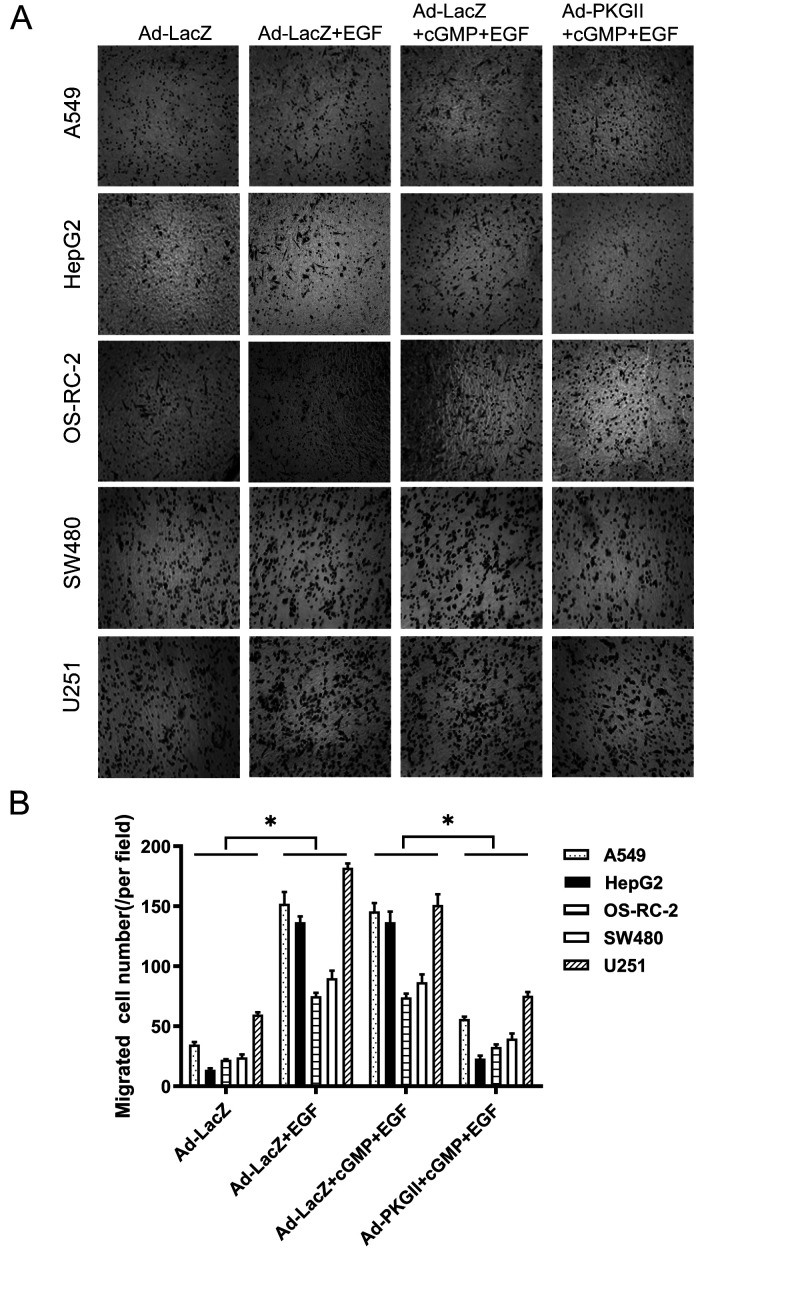
PKG II overexpression inhibits cancer cell migration. The migratory capabilities of human A549 lung, HepG2 hepatic, OS-RC-2 renal, SW480 colon cancer and U251 glioma cells were examined using a Transwell assay. Cells were infected with Ad-LacZ or Ad-PKG II for 36 h, serum-starved overnight, stimulated with 8-pCPT-cGMP (250 µΜ) and EGF (100 ng/ml), and cell migration was analyzed after 12 h. (A) Representative photomicrographs of migrated cells following Giemsa staining (magnification, 50×). (B) The number of migrated cells in the various treatment groups. Data are presented as the mean ± standard deviation of 3 independent experiments. *P<0.05, comparison between groups as indicated by lines. PKG II, cGMP-dependent protein kinase type II; Ad-LacZ, adenoviral vector containing the β-galactosidase gene; Ad-PKG II, adenoviral vector containing the PKG II gene; 8-pCPT-cGMP, 8-(4-chlorophenylthio)-cGMP; cGMP, cyclic guanosine monophosphate; EGF, epidermal growth factor.

**Figure 6.**
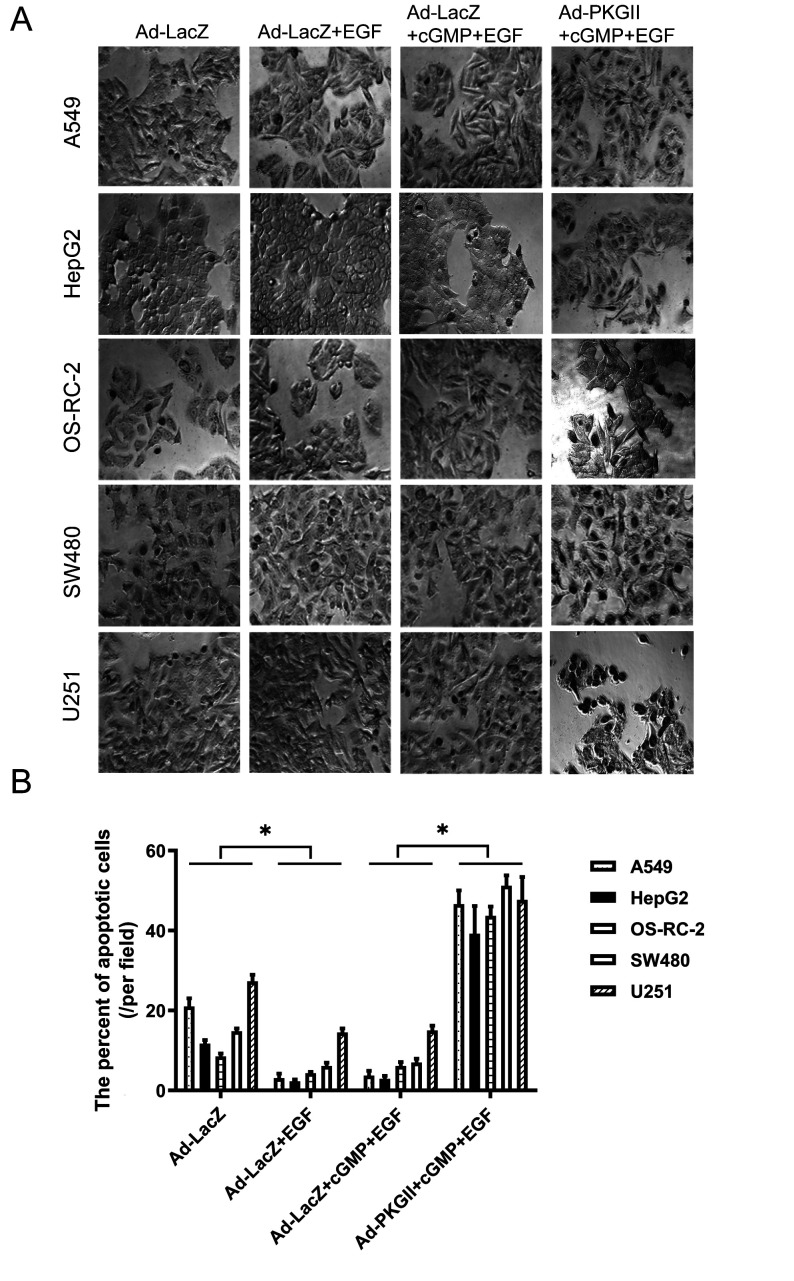
PKG II overexpression counteracts the anti-apoptotic effects of EGF. A terminal deoxynucleotidyl transferase 2’-deoxyuridine, 5’-triphosphate nick-end labeling assay was performed to detect apoptotic cells among human A549 lung, HepG2 hepatic, OS-RC-2 renal, SW480 colon cancer and U251 glioma cells. (A) Representative photomicrographs of apoptotic cells under ×200 magnification. (B) The percentage of apoptotic cells/field of view in the various treatment groups. Data are expressed as the mean ± standard deviation of 3 independent experiments. *P<0.05, comparison between groups as indicated by lines. PKG II, cGMP-dependent protein kinase type II; EGF, epidermal growth factor; Ad-LacZ, adenoviral vector containing the β-galactosidase gene; Ad-PKG II, adenoviral vector containing the PKG II gene; cGMP, cyclic guanosine monophosphate.

